# A. V. M. A.

**Published:** 1900-05

**Authors:** 


### A. V. M. A.

Secretary Stewart has just issued the following important letter in connection with the coming thirty-seventh annual meeting:

At Detroit, Mich., on September 4,5,6,and 7,1900, the American Veterinary Medical Association will hold its thirty-seventh annual meeting. The working motto of this Association is usefulness. It aims to develop and disseminate tangible, helpful thought and knowledge in the veterinary profession throughout the continent of America. Year by year its sphere of influence becomes more and more wide-spread, and its potency for advancing and enlarging public knowledge and appreciation of veterinary science as a useful and indispensable factor in the promotion of public welfare is more clearly recognized. The substantial character of the progress it is making is noted at home and abroad. The papers presented and discussed at its meetings merit and receive the careful attention of medical men of other continents. English veterinary editorials call attention to the great value of the published reports of the proceedings of this Association, and urge English Associations to emulate its example.

In addition to the regular meeting of the A. V. M. A. there will be held meetings of the Association of Experiment Station Veterinarians and the Association of Veterinary Faculties and State Boards of Veterinary Examiners.

The difficulties encountered in the enforcement of municipal and State sanitary regulations must always engage the serious attention of veterinarians. A constantly increasing number of them are employed in the control of hog-cholera, glanders, tuberculosis, and other diseases, slaughter-house and market inspection, and milk and dairy inspection. New problems or new phases of old problems are constantly developing. A number of papers relating to this phase of sanitary medicine will be included in the programme.

The clinics held in connection with the last two meetings have proved so interesting and instructive that this feature of the programme will be given more time during the coming meeting. It is probable that there will be three clinical sessions, and a considerably larger number of operations demonstrated. The practical value of the clinic is no longer questioned. The explanatory remarks made in connection with each surgical demonstration and the numerous papers relating to general practice must certainly appeal very strongly to every practising veterinarian.

The Committee on Local Arrangements has its plans about completed, and you are assured that nothing will be lacking to make the social phase of our meeting a most pleasant memory. Detroit is famous for its hospitality, and members and visitors who attend this meeting of the A. V. M. A. will certainly join with the hosts who sing its praises.

The number in attendance at the meeting in New York City last year was fully double that of any previous meeting. Over sixty ladies accompanied the members and visitors. They participated in and added greatly to the social pleasures of the meeting. Of those who attended this meeting the one has yet to be found who did not realize that he was many times repaid for his expenditure of time and money. The programme for the meeting to be held in Detroit will be a very attractive one, and it is confidently expected that the attendance will be even larger than last year. Who can afford to stay away if it is possible to attend? This timely notice is given that you may plan to be present.

The following proposed amendments to the Constitution will come before the Association for its consideration and adoption or rejection :

To amend Article III. of the Constitution by omitting the words "three Vice-Presidents, one from the East, one from the Central, and one from the Western section of the country," and add after the "President" the words "five Vice-Presidents."

The present state of the treasury permits the prompt publication of the proceedings of our meetings, and the Publication Committee was enabled to place the report of the last meeting in your hands withn sixty days after the close of the New York meeting. Many letters expressing appreciation of this report, both for its prompt publication and the fulness of detail, have been received. The Committee welcomes criticism of this report as well as suggestions intended to increase the value of the report of the meeting to be held in Detroit.

If you intend to prepare a paper for the programme of the Detroit meeting, and you are respectfully urged to do so, you should notify me at once, stating title of same.

If there be any errors in the address on the enclosing envelope kindly advise me of the same, that our books may be corrected and make certain the delivery of future communications from this office.

If your neighbor veterinarian is likely to be interested in the work of this Association and would probably apply for membership if solicited, will you not write to the resident Secretary of your State and secure an application blank and copy of By-laws for your neighbor's consideration?

Your attention is respectfully called to the enclosed statement of your account for dues, and you are urged to give this matter early attention. You know upon reflection that prompt payment of dues constitutes an elixir of life to an organization. Your proportion of said elixir is needed to maintain the vitality of the A. V. M. A.

